# Patient-defined desired outcome, success criteria, and expectation in outpatient physical therapy: a longitudinal assessment

**DOI:** 10.1186/s12955-017-0604-1

**Published:** 2017-01-31

**Authors:** Giorgio Zeppieri, Steven Z. George

**Affiliations:** 1 UF Health at the Orthopedic and Sports Medicine Institute, 3450 Hull Road, Gainesville, Fl 32607 USA; 2 Department of Orthopeadic Surgery and Duke Clinical Research Institute, Duke University, PO Box 17969, 2400 Pratt Street, Room 0311 Terrace Level, Durham, North Carolina USA

**Keywords:** Orthopedics, Patient-Centered outcomes, Patient satisfaction, Physical Therapy

## Abstract

**Background:**

Patient-centered approaches offer an alternative method in evaluating treatment outcomes. This study investigated; 1) if patient’s criteria for success (satisfaction of clinical outcomes) changes from pre to post treatment, 2) whether patients who met their success criteria also meet minimal clinical important difference scores (MCIDs), and 3) if patient’s success criteria differed from their expected (what the patient believes will occur) and desired (what the patient wants to occur) outcomes following intervention.

**Methods:**

A consecutive sample of 225 subjects with complaints of musculoskeletal pain was referred to an outpatient, sports medicine physical therapy clinic. Participants completed the Patient Centered Outcome Questionnaire (PCOQ) prior to their initial evaluation session and a follow-up PCOQ at discharge. The PCOQ asks subjects to rate their pain, fatigue, emotional distress, and interference with daily activities for usual, desired, successful, and expected levels, and how important improvement is for each domain on a 101-point numerical rating scale. Paired-sample *T*-test were used to determine patient’s pre and post success criterion and whether success criteria differed from desired and expected outcomes following intervention. Chi-squared were used to determine if individuals desired, expected, and success criteria for treatment outcome differed from established MCIDs.

**Results:**

The results revealed no change in success criteria pre to post treatment for all domains. Chi-square test revealed patients desired, expected, and success criteria were independent of established MCIDs (*P >* .01). There were no differences between patients expected outcomes and success criteria. However, there were differences between patient’s desired outcomes and expected and success outcomes, with patients reporting lower desired levels of pain, emotional distress, fatigue, and interference with daily activities following physical therapy intervention (*P <* .01).

**Conclusion:**

Patients in this setting do not appear to modify their success criteria throughout the course of outpatient physical therapy. Additionally, individually defined success criterion differs from established clinically important changes. Clinicians interested in a broader assessment of outcome need to consider patient determined criterion in addition MCIDs. Furthermore, desired outcomes are lower than both expectation and success criteria. In this setting, outcomes following physical therapy episodes were likely to meet patient’s expectations and success criteria but not desired criterion.

**Electronic supplementary material:**

The online version of this article (doi:10.1186/s12955-017-0604-1) contains supplementary material, which is available to authorized users.

## Background

There has been an increased awareness in the patient’s perspective when evaluating outcomes in clinical practice. A patient-centered approach emphasizes patient’s desires, beliefs, and expectations and utilizes them in making decisions on what constitutes a successful treatment outcome [1]. A model with the patient as the centerpiece of outcome driven decisions may have advantages when assessing treatment outcomes. Patient-centered models allow patients more control in directing their treatment and are a viable alternative method in determining the success of treatment outcomes [1–6]. A patient-centered approach has the potential to lead to increased satisfaction, enhanced patient-practitioner interaction, greater treatment compliance, and a positive treatment response [4–7].

As patients perspective of their state of musculoskeletal dysfunction changes, so will their definition of what an acceptable outcome should be following treatment [8–10]. This change in patient’s self-evaluation of outcome criterion represents a response shift [8–10]. This psychological phenomenon occurrs during the course of treatment as patients adapt and attain knowledge of their musculoskeltal condition [10]. Recognizing that patient’s self evaluation of treatment outcome may change during the course of treatment may lead to more accurate interpretations of the effectiveness of rehabilitation [10].

Patients seeking physical therapy interventions have individualized markers of success for desired and expected outcomes [11, 12]. Clinical outcomes are influenced by patients *desired*, *expected*, and *successful* criterion [11–13]. They can be mistaken as similar paradigms, but in fact influence outcomes differently [11, 13]. For instance, a patient’s desired outcome criteria are indicative of what patients want to occur in a best case scenario following an intervention [11, 13–16]. Therefore, desired criterion may or may not reflect a realistic treatment outcome depending on the condition being treated [13, 17]. Patient’s expected outcome criteria indicate what patients believe will occur following interventions with previous studies indicating patient expectations have the potential to influence outcomes both positively and negatively [11, 13, 18–24]. Finally, patients’ successful outcome criteria are an indication of patient’s beliefs of what should occur following therapy that would result in satisfaction or dissatisfaction with outcome [5, 11, 13, 25, 26]. In contrast with desired and expected outcomes, a successful outcome is a measure of a patient’s essential mimimum level of achievement in order to judge an outcome successful.

An understanding of the differences between these three mediators of outcome are important in evaluating the success of outcomes from a patient centered perspective and also serve to maximize treatment outcomes [11, 13]. The influences of the three mediators act as prognostic indicators to clinical outcomes and patient satisfaction with treatment episodes, which may be stronger than the treatment effect itself on outcomes (13). Each mediator may potentially influence clinical outcomes differently such as in functional reports and satisfaction of care but are not always taken into account in clinical practice. Furthermore, when they are accounted for in clinical practice only one of these mediators is typically measured, and a comprehensive assessment may be necessary to better appreciate the patient perspective. Also, an expectation for a specific treatment outcome may help identify best treatment practice for patients [13]. Additionally, practioners should be able to distinguish practical from unrealistic outcomes, when treating patients and be able to direct the patient towards a more likely feasible and reasonable treatment outcome [13, 17].

Our first objective was to investigate if patient’s success criteria changed from pre to post treatment. Furthermore, we also investigated whether patients met their success criteria and what amount of change was necessary for a treatment outcome to be rated as successful. We hypothesized that patients would not meet their own success criteria, as it has been shown that patients require greater improvements in outcomes than what is typically achieved [2, 3]. Our second objective was to examine whether patients who met their success criteria also met minimal clinical important difference scores (MCIDs) in orthopedic and sports medicine rehabilitation practice. We hypothesized based on our previous work that patient centered outcomes may require greater improvement than what has previous been reported as clinically meaningful [3]. Our third objective was to investigate if patient’s success criteria differed from their expected and desired criteria. We hypothesized that these mediators may be theoretically different constructs, which patients use to judge clinical outcomes.

## Methods

### Participants

A consecutive sample of 225 subjects (126 male, 99 female) referred to outpatient, sports medicine physical therapy with complaints of musculoskeletal pain and decreased function was recruited over the course of one year by multiple therapists (*n =* 5). This sample was for a planned follow up study and was recruited independently from our previously reported study [3]. Patients were included in this analysis if they presented with an orthopedic condition that was appropriate for physical therapy. Appropriateness for physical therapy was determined by medical diagnosis and history. No patients were excluded from this sample population based on this criterion and 204/225 (90.6%) patients returned for follow-up and completed the discharge assessment.

### Procedure

At the initial intake physical therapy session, subjects completed the Patient Centered Outcome Questionnaire (PCOQ) (Additional file 1), which obtains the patient’s perspective of treatment goals across four constructs (pain, fatigue, emotional distress, and interference with daily activities) and assesses how each of these areas has impacted the patient and what the patient’s definition of expected, successful, and desired outcomes are following their rehabilitation treatment [1–4]. Additionally, subjects completed a standard medical and demographic questionairre, as well as outcome measures matched to patient presentation and appropriate for region of symptoms, the Tampa Scale for Kniesophobia-11 (TSK-11), and the Short Form of the Medical Studies −8 (SF-8). All questionairres were given out as part of routine clinical care and patient-assessment, prior to initiation of treatment. Patients also completed the Follow-up PCOQ, TSK-11, SF-8 and region specific outcome measure at discharge. In addition, all patients treated at the University of Florida Orthopeadic and Sports Medicine Institute sign a global IRB approved by the University of Florida Institutional Review Board prior to initiating treatment. This global IRB allows for use of data from questionnaires related to injury that measure pain, functional, and psychological outcomes in a de-identified manner. The de-identified clinical data were then entered into a clinical data bank before analysis.

### Patient centered outcome measure

The PCOQ is comprised of five sections with four domains (pain, fatigue, emotional distress, and interference of daily activities) for usual, desired, successful, and expected levels, as well as how important improvement is for each domain on a 101-point numerical rating scale (NRS; 0 = none, 100 = worst imaginable) [27]. In each section, the subject is asked to rate their level of pain, fatigue, emotional distress, and interference of daily activities on a 101-point NRS for their usual, expected, and desired level, as well as what treatment outcome they would consider successful. The subject is then asked to identify what they expect following their physical therapy intervention and how important they consider an improvement in each of these domains [1–4, 27]. In previous studies, the PCOQ has shown a test-retest reliability over a 48-h period of ICC = .84 to .90 (*P <* .001) for usual levels of pain, fatigue, distress, and interference with daily activities, as well as ICC = .43–.58 (*P <* .05) for success criteria in all domains with the exception of emotional distress ICC = .29 (*P =* .21) [1, 4]. Additionally, the PCOQ has demonstrated good concurrent validity with measures associated with pain and disability [1, 4].

The Follow-up PCOQ (Additional file 2) is comprised of 6 sections with four domains (pain, fatigue, emotional distress, and interference with daily activities) for usual pretreatment, successful, important, and usual post treatment levels for each domain on a 101-point numerical rating scale (NRS; 0 = none, 100 = worst [1]. Then, the patient is asked whether their condition improved, stayed the same, or worsened in each domain since beginning physical therapy and how much they improved or worsened on a 101-point NRS [1]. Lastly, patients indicate if physical therapy interventions were successful in each domain by choosing yes or no, and whether their overall treatment was successful (yes or no) [1].

Generic Measures (Each generic measure was completed by all patients regardless of location of orthopedic condition). These measures are described in more detail below:

### Pain intensity

The numeric pain rating scale (NPRS) is an 11-point, self-report rating scale that askes individuals to assess the intensity of their current pain on a scale from 0 (no pain) to 10 (worst pain imaginable) [28–30]. The NRS test-retest reliability in patients has been shown to be moderate (ICC = .74) and the MCID has been reported to be 2.2 in patients with shoulder pathology [28–30].

### Quality of life (QofL)

The short form of the medical studies 8 (SF-8) is an 8-item health related quality of life survey adapted from the SF-36. The SF-8 has two subscales; a physical (PCS) and mental (MCS) health scale. Questions measure general health, physical role, bodily pain, vitality, social functioning, mental health, and emotional role. The items are scored on an ordinal scale and point assessment is based on the number of selections possible [31]. Lower scores indicate better health and a higher quality of life, while higher scores indicate poorer health and a lower quality of life. The SF-8 has shown a reliability of (ICC > .61) for PCS and (ICC > .68) for MCS [31]. The SF-8 MCID is 10 points for both the PCS and MCS [31].

### Fear of movement

The Tampa Scale for Kniesophobia-11 (TSK-11) is an 11-item shortened version derived from the original TSK-17 [32]. The questionnaire assesses an individual’s pain-related fear of movement on a 4-point Likert scale (1-strongly disagree, 2- somewhat disagree, 3-somewhat agree, 4-strongly agree) [28, 32]. Each item is scored and then summed. Higher scores indicated higher pain-related fear of movement, while lower scores indicated lower pain-related fear of movement [28]. The questionnaire was originally intended to assess pain-related fear in a chronic low back pain population, but has been recently used to assess pain-related fears in other movement related orthopedic dysfunctions [28]. The TSK-11 has good internal consistency (Cronbach’s alpha=. 79), test-retest (ICC=. 81, SEM = 2.54), responsiveness (standardized response mean (SRM) = −1.11) and an MCID of 4.8 [28, 32, 33].

### Region specific measures

The shoulder pain and disability index (SPADI) is a 13-item questionnaire that is comprised of two outcome subscales (pain and disability) [34, 35]. The pain subscale consists of 5-items, while the disability subscale consists of 8-items. Each subscale is assessed on an 11-point numeric rating scale (NRS). The patient is asked to circle a number 0 (“no pain at all” or “no difficulty”) through 10 (“worst pain imaginable” or “so difficult it required help”) that best corresponds to their present state [35–37]. Each subscale is then averaged to give a total score out of 100. Higher scores define higher dysfunction. The SPADI has good reliability (ICC ≥ .89) and good responsiveness to change over time [36]. The MCID is 8 points, which represents the smallest detectable change that is important to the patient [37].

The International Knee Documentation Committee Subjective Knee Form (IKDC) is an 18-item questionnaire, which assess an individual’s knee function. Items are scored based on total possible selections present. For example, an item that contains two possible selections would be scored 0–1; where as an item with five possible selections would be scored 0–4. All items are then summed to determine the raw score, then divided by the total score possible, and finally multiplied by 100 [28, 38]. Higher scores are equated with higher knee function, while lower scores indicate lower knee function. The IKDC has shown good reliability (ICC =. 94) and responsiveness (SRM = .94) [23, 28, 39, 40]. The MCID of the IKDC has been reported as 3.19 points [41].

The Oswestry Disability Index (ODI) is a 10-item questionnaire that assesses how back dysfunction affects an individual’s pain and functional activities (personal care, lifting, walking, sitting, standing, sleeping, social life, traveling, and employment) on 6-point numerical scale (0–5) [42–44]. Each section is scored and then summed. The summed score is then divided by 50, which is the total possible score, and multiplied by 100. A score of 0-20% is equated to “minimal disability”, 21-40% to “moderate disability”, 41-60% to “severe disability”, 61-80% to “crippled”, and 81-100% to “bed-bound” or “exaggeration” [42–44]. The ODI has shown good reliability within the literature (ICC ≥ .78 to ICC ≥ .90) and an area under the curve (ROC) index of .76 [42–48]. The MCID for the ODI is 10 points [42, 43, 48].

The lower extremity functional scale (LEFS) is 20-item questionnaire that assess an individual’s lower extremity dysfunction on a 5-point Likert scale [49]. The patient is asked to circle a number from 0–4, where 0 corresponds to “extreme difficulty” or “unable to perform”, 1 to “quite a bit of difficulty”, 2 to “moderate difficulty”, 3 to “a little bit of difficulty”, and 4 to “no difficulty”. The choices are then summed and divided by 80 (total possible score) [49]. Lower scores indicate a higher level of disability, while higher scores indicate lower levels of disability [49, 50]. The LEFS has excellent reliability (ICC = .98) and also found to have good construct validity (*r* = .80) [49, 50]. The LEFS MCID is 9 points [49, 50].

### Operational definition

MCID was defined as the smallest difference in score in a domain of interest or outcome that could be defined as beneficial based on an external criterion [51]. Previously established MCID’s were used for the measures included in this study.

### Data analysis

All analyses were performed with SPSS version 20.0 (Chicago, IL) with a α = .01 due to the number of comparisons. Descriptive statistics were calculated for selected demographic and clinical factors and reported as mean and standard deviation for continuous variables or frequency for categorical variables. Normality of PCOQ domains was assessed with one-sample Kolmogorov-Smirnov tests.

### Patient centered outcome questionnaire changes from Pre to post treatment

Change scores in each PCOQ domain (PCOQ initial ratings- Follow-up PCOQ usual ratings) were calculated. Paired samples-*T* test were performed to determine if subjects mean success criterion differed between pre and post-treatment in each domain. Additionally, interclass correlation coefficients (ICC) for pre to post change scores were calculated to determine agreement for pre and post scores. Success criterion “met or not met” in each PCOQ domain was also calculated (Post treatment usual levels—Pretreatment success score).

### Comparisons with previously established MCIDs

Chi-Squared test were used to determine if individual success criteria for treatment outcome differed from accepted MCIDs for each of the region specific and generic measures (group criteria).

### Differences between patient defined desired, expected, and successful outcomes

Desired and expected change was calculated by subtracting subject’ desired and expected levels of impairment following treatment from subjects’ usual levels of impairment at initial intake. One-way ANOVA with Scheffe post-hoc testing were also used to assess whether subject’s success criteria differed from their expected and desired outcomes.

## Results

Descriptive statistics for sample demographics are listed in Table 1.

**Table 1:** Demographic and clinical data for the total sample

Variable	Total Sample (*n =* 225)
Age, mean years (SD)	32.8 (16.7)
Sex	
# Male (%)	126 (56%)
# Female (%)	99 (44%)
Race	
# White (non-Hispanic)(%)	174 (77.3%)
# Hispanic (%)	15 (6.7%)
# African American (%)	24 (10.7%)
# Asian (%)	11 (4.9%)
# Arabic (%)	1 (.4%)
Anatomical location of symptoms	
# Cervical Spine (%)	3 (1.3%)
# Thoracic Spine (%)	1 (.4%)
# Shoulder (%)	71 (31.6%)
# Elbow/Wrist/Hand (%)	6 (2.7%)
# Lumbar Spine (%)	21 (9.3%)
# Hip (%)	2 (.9%)
# Knee (%)	113 (50.2%)
# Foot/Ankle (%)	8 (3.6%)
Duration, mean days (SD)	53.0 (220.9)
# Acute (%)	119 (52.9%)
# Sub-acute (%)	76 (33.8%)
# Chronic (%)	30 (13.3%)
Pain level at initial evaluation	
# Current level (SD)	3.9/10 (2.5)
# Worst level (SD)	7.5/10 (2.3)
# Best level (SD)	2.6/10 (2.3)
Post-operative	
# Yes (%)	64 (28.6%)
# No (%)	160 (71.4%)
Contact Injury	
# Yes (%)	61 (27.1%)
# No (%)	164 (72.9%)
Traumatic Injury	
# Yes (%)	109 (48.4%)
# No (%)	116 (51.6%)
Prior Physical Therapy	
# Yes (%)	91 (40.4%)
# No (%)	134 (59.6%)
Prior Physical Therapy Intervention Successful	
# Yes (%)	68 (74.7%)
# No (%)	23 (25.3%)
Prior Other Healthcare	
# Yes (%)	44 (19.6%)
# No (%)	181 (80.4%)
Prior Other Healthcare Successful	
# Yes (%)	33 (75%)
# No (%)	11 (25%)
Exercise Regularly Prior To Injury	
# Yes (%)	122 (54.5%)
# No (%)	102 (45.5%)

Note: Values are number (%) or as otherwise indicated

### Changes from patient centered outcome questionnaire Pre to post treatment

Results for PCOQ and Follow-up PCOQ mean domain levels for usual, successful, and importance outcomes are listed in Table 2. Subjects reported their mean usual levels of pain, fatigue, emotional distress, and interference with daily activities before treatment (Mean = 25.7–46.7). Post-treatment subjects reported a lower level of usual pain, fatigue, emotional distress, and interference in daily activities (Mean = 12.4–21.8) (NRS; 0 = none, 100 = worst imaginable). Descriptive statistics for subjects who “met” or “did not meet” their initial success, expected, or desired criterion following physical therapy intervention are listed in Table 3.

**Table 2:** Usual, desired, expected, and successful outcome domain distributions for total data distributions

Domain	Level	Pretreatment Mean^a^ (SD)	Post-treatment Mean^a^ (SD)	t	P
Pain	Usual Levels	42.1 (28.1)	21.3 (23.5)	11.4	.001
	Desired Levels	5.2 (14.2)			
	Expected Levels	11.8 (18.5)			
	Success Levels	13.9 (18.8)	14.6 (23.3)	–.34	.735
Fatigue	Usual Levels	31.5 (27.3)	17.8 (22.9)	8.1	.001
	Desired Levels	5.5 (12.9)			
	Expected Levels	12.7 (18.7)			
	Success Levels	13.9 (17.6)	12.5 (20.6)	.71	.482
Emotional Distress	Usual Levels	25.7 (27.1)	12.4 (20.1)	8.6	.001
	Desired Levels	4.0 (11.5)			
	Expected Levels	8.3 (15.8)			
	Success Levels	9.3 (15.4)	9.5 (19.7)	–.09	.932
Interference with Daily Activities	Usual Levels	46.7 (31.3)	21.8 (25.4)	9.7	.001
	Desired Levels	6.2 (19.3)			
	Expected Levels	10.3 (18.8)			
	Success Levels	12 (19.5)	10.6 (20.9)	.72	.476

^a^Numerical rating scale = 0–100

**Table 3:** Descriptive data for subjects who met or did not meet initial self-report criterion for success, expected, and desired outcomes following physical therapy intervention

PCOQ Domain	Success Criterion Met/Not Met	Expected Criterion Met	Desired Criterion Met
Pain	Met: 182/225 (80.9%)	Met: 126/225 (56%)	Met: 83/225 (36.9%)
	Not Met: 43/225 (19.1%)	Not Met: 99/225 (44%)	Not Met: 142/225 (63.15%)
Fatigue	Met: 137/225 (60.9%)	Met: 119/225 (52.8%)	Met: 103/225 (46%)
	Not Met: 88/225 (39.1%)	Not Met: 106/225 (47.2%)	Not Met: 122/225 (54%)
Emotional Distress	Met: 121/225 (53.8%)	Met: 155/225 (68.8%)	Met: 115/225 (51%)
	Not Met: 104/225 (46.2%)	Not Met: 70/225 (31.25%)	Not Met: 110/225 (49%)
Interference with Daily Activities	Met: 176/225 (78.2%)	Met: 122/225 (54%)	Met: 93/225 (41.3%)
	Not Met: 49/225 (21.8%)	Not Met: 103/225 (46%)	Not Met: 132/225 (58.7%)

Note: *PCOQ* Patient Centered Outcome Questionnaire

Paired-samples *t* test results revealed a decrease in their usual levels of pain, fatigue, emotional distress, and interference in daily activities (*P <* .001). The results revealed no change in treatment success criteria pre to post treatment for all domains; pain (t = −3.4, df = 203, *p*=. 74), fatigue (t=. 71, df = 203, *p*=. 48), emotional distress (t = −.09, df = 203, *p =* .93), and interference with daily activities (t = .72, df = 203, *p =* .48). These results indicated that subject’s target values for treatment success do not change during the course of physical therapy treatment (*p* ≥ 0.01).

Agreement for pre to post change scores was low across all domains; pain (ICC = 0.13, F = 1.302, *p =* .03), fatigue (ICC = 0.18, F = 1.448, *p =* .004), emotional distress (ICC = .17, F = 1.420, *p =* .006), and interference with daily activities (ICC = .20, F = 1.506, *p =* .002).

### Comparisons with previously established MCID’s

Descriptive statistics for generic and region specific measures are listed in Table 4. Chi-square test revealed patient’s individual desired, expected, and success criteria were independent of the accepted MCID for all region specific measures used in this study (*P >* .01).

**Table 4:** Descriptive data for region specific and generic questionnaires and percentage of sample that met MCIDs verse individual success criteria

Questionnaire	Pre Treatment Mean (SD)	Post Treatment Mean (SD)	Mean change	% MCID Met	% Individual Success Criteria Met
NPRS	3.87/10 (2.1)	2.7/10 (1.7)	1.17 points	Met: 98/201 (48.7%)	^a^Met: 43/201 (21.4%)
SPADI	50.3/100 (25.5)	29.6/100 (24.5)	20.7 points	Met: 49/66 (74.2%)	^b^Met: 21/66 (31.8%)
IKDC	44.1/100 (21.7)	63.9/100 (17.8)	19.9 points	Met: 64/106 (60.4%)	^b^Met: 12/106 (11.3%)
Oswestry	23.2/100 (17.1)	14.5/100 (15.9)	8.7 points	Met: 1/16 (6.3%)	^b^Met: 1/16 (6.3%)
LEFS	53.8/80 (24.6)	75.8/80 (25.2)	22 points	Met: 7/9 (77.8%)	^b^Met: 2/9 (22.2%)
SF-8 (Mental)	51.3 (9.6)	53.8 (8.5)	2.5 points	Met: 34/201 (16.9)	^c^Met: 29/201 (14.4%)
SF-8 (Physical)	40.3 (9.6)	47.9 (8.2)	7.6 points	Met: 84/201 (41.8%)	^d^Met: 35/201 (17.4%)
TSK	24.4/44 (6.3)	19.2/44 (6.0)	5.2 points	Met: 107/200 (53.5%)	^c^Met: 79/200 (39.5%)

Note

*NPRS* Numerical Pain Rating Scale, *SPADI* Shoulder Pain and Disability index, *IKDC* International Knee Documentation Committee Subjective Knee Form, *LEFS* Lower Extremity Functional Scale, *SF8* Short Form of the Medical Studies −8, *TSK* Tampa Scale for Kniesophobia-11

^a^% of Individuals who met PCOQ Pain Domain Success Criterion

^b^% of Individuals who met PCOQ Interference with Daily Activities Success Criterion

^c^% of Individuals who met PCOQ Emotional Distress Success Criterion

^d^% of Individuals who met PCOQ Fatigue Success Criterion

### Differences between patient defined desired, expected, and successful outcomes

**Fig. 1:**
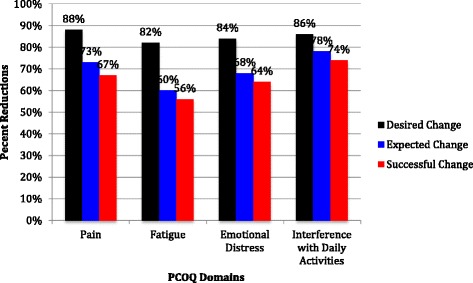
Percent reductions, required in desired, expected, and successful outcomes by patients in pain, fatigue, emotional distress, and interference in daily activities

There were no differences between expected outcomes of pain fatigue, emotional distress, and interference with daily activities following PT interventions and their success criteria (*P >* .01). There were differences between patient’s desired outcomes for pain, fatigue, emotional distress, and interference with daily activities and their expected outcomes. Additionally, there were also differences between patient’s desired outcomes for pain, fatigue, emotional distress, and interference with daily activities and their success criteria (*P <* .01) (Fig. 1).

## Discussion

This study is the first longitudinal study we are aware of to define patient determined success criteria beyond the minimum level of change in an outpatient sports medicine rehabilitation clinic. Patient-centered perspectives regarding treatment success are important to explore because they are linked with improved patient satisfaction and encourage focus on clinical outcomes that “matter most to patients” [52, 53]. Additionally, this study described differences between patient’s desired, expected, and successful outcomes when seeking physical therapy intervention. These analyses indicated that there were differences between patient’s desired rehabilitation outcome criteria and their expected and satisfactory outcomes criteria following outpatient physical therapy. Furthermore, patient-centered desired treatment outcomes were more stringent than what patients may consider a successful or expected outcome following physical therapy. These differences can be used to direct the patient towards pragmatic outcomes as they can elucidate unrealistic expectations from likely treatment outcomes and minimize patient dissatisfaction [4]. For example, a very active 45-year-old female runner with a medical diagnosis of intra-articular hip joint pathology is referred to physical therapy for strengthening and manual therapy. The patient may desire to be pain free and return to the same high level of running without symptoms, however these outcomes may be unlikely depending on the severity of the intra-articular pathology in this particular case. The desired outcome is an absolute decision for the patient (a optimal outcome to be pain-free), but relative for the clinician (they can only offer what treatments are available). Physical Therapy should incorporate interventions to educate the patient in how to manage their pain, supplementing running mileage with non-impact activities such as swimming and biking, as well inform the patient that a more probable outcome may be a partial reduction in pain with an increase in functional activities.

The ability to determine patient-centered success criteria may assist the therapist towards aiding the patient in achieving clinical outcomes that are likely in scope [3]. Based on our findings, patients seeking physical therapy intervention in an outpatient, sports medicine, orthopedic setting do not modify their absolute outcomes criteria for success in selected outcome domains, despite changes in usual levels of pain, interference, emotional distress, and fatigue. Although mean scores for pre to post treatment were not different, agreement was low which may suggest that the time point tested may matter as success criteria may be influenced by patient’s current status and is subject to vacillation. Further longitudinal research is necessary to investigate the stability of success criteria and determine if the low agreement is due to the drift, whether subject’s success criterion differed from pre to post treatment as individuals’ positive changes and negative changes cancel each other out.

Our results replicate prior findings that patient defined successful outcomes are not influenced by demographic data. These results would indicate an absence of need of specific region measures to determine patient defined treatment outcomes, since these outcomes were not influenced by specific physiological or demographic factors. These findings seem counter-intuitive due to the heterogeneity of patients seeking outpatient physical therapy, however it seems that patient’s outcomes, whether they be ideal, expected, or successful are specific to the shared circumstance of the attainment of clinical goals (i.e. decrease in pain, fatigue, emotional distress, and increase in the ability to perform ADLs) [13].

Patient’s individual criterion for treatment success was independent of MCIDs for region specific and generic measures. Our data indicate that patient defined definitions of outcome required greater improvement in selected domains than commonly accepted MCIDs in region specific, fear of re-injury, and quality of life questionnaires. This is an indication that MCIDs should not be the only determinant for the effectiveness of clinical interventions. Considering patient determined criteria for success might provide greater insight to outcome assessment, especially for those interested in patient defined criteria [2, 3]. Specifically, our study indicates that clinicians should not rely solely on MCIDs to determine treatment outcomes, since patient centered definitions of success may differ. In fact, based on these results the MCIDs seem to underestimate the amount of change that is needed to reach patient defined goals for expected, successful, and desired outcome levels.

The findings also elucidate the differences between patient’s desired, expected, and successful outcomes prior to physical therapy intervention. The data suggests that expected and success outcomes may be different constructs, but patients report similar outcome values, however the data also indicate that desired outcome criteria are different than both expected and successful outcome criteria. Patients may have very stringent criteria when entering rehabilitation following injury in what they want to occur following physical therapy intervention [13]. The data supports this association because there were similar expectations for treatment success following rehabilitation, but their success criteria and expectations never reached desired or ideal outcome. This may account for the fact that patients still seek further reductions in pain, fatigue, emotional distress, and limitations in ADLs at discharge, despite reporting satisfaction with outcomes. We speculate that the inability to meet desired outcome levels could be the reason some patients seek additional health care after physical therapy. It is important to note that these findings are not indicative of failure to meet patient pretreatment expectations or satisfaction, but in fact an indication of a failure to meet the desired outcome (i.e. a different, more stringent construct). It is also important to note that practitioners should determine what patients expectations are during the initial intervention in order to ascertain if the patient’s expectation is pragmatic or not and provide education to a more practical expectation. It is necessary for clinicians to educate patients that desired levels could be difficult to achieve, which may lead patients to re-rank the importance of selected outcomes, make informed decisions, as well as contribute to more practical outcomes [4].

Additional research should look into the importance of determining whether treatments that provide desired levels of treatment outcome equate to patients using less health care in the future and therefore potentially decreasing healthcare cost and potentially reducing the burden on the healthcare system.

### Study limitations

This study has several limitations, which should be considered when interpreting the results. One limitation is that our findings are most applicable to outpatient orthopedic and sports medicine population and may not be indicative of other physical therapy inpatient or outpatient populations. Other populations may have different inherent definitions of success, desired and expected outcomes. Furthermore, our study cannot differentiate if patient’s expectations changed differentially based on specific physical therapy interventions as we just considered their general response to receiving physical therapy services. Additionally, our study examined pre to post changes in patient success criteria, but did not measure pre to post changes in desired or expected outcome criteria. We are unable to accurately say if patients modify their expected or desired outcome criteria based on improvements in outcomes or if they make judgments on the effectiveness treatment interventions based on their pretreatment definitions of outcome expectation. Furthermore, our findings are most relevant to a patient population defined by an equal distribution of traumatic verse non-traumatic injuries, with 27% presenting with contact injuries. Our results cannot be indiscriminately, generalized to other populations or populations with chronic orthopedic conditions. Also, the questionnaires are given to all patients at their first physical therapy visit prior to initiation of treatment and as part of routine clinical care, however we did not collect data on the number of patients who refused or did not complete the questionnaires. We cannot speculate that those patients who did not complete the questionnaires would report similar criteria for success than those who did.

## Conclusions

This current study confirmed our results from our previous study that patients may report greater improvements in treatment outcomes when using a patient centered measure as compared to what has been previously reported as clinically meaningful [3]. Furthermore in this setting, patients do not modify their absolute success criteria throughout the course of physical therapy treatment. Clinicians may want to consider that MCIDs are patient outcomes that determine the minimum level of outcome needed and do not define patient’s maximum amount of change needed to judge an outcome successful. Patient’s expectations for treatment outcomes for pain, fatigue, distress, and interference with ADLs are not different from patient’s success criteria, but their desired outcomes are more stringent.

## Additional files

Additional file 1: Patient Centered Outcome Questionnaire. (DOCX 11 kb)
Additional file 2: Follow-up Patient Centered Outcome Questionnaire. (DOCX 14 kb)
