# Supplementary material for: Patient-defined desired outcome, success criteria, and expectation in outpatient physical therapy: a longitudinal assessment
Source: Health Qual Life Outcomes. 2017 Jan 31;15:29. doi: 10.1186/s12955-017-0604-1 (PMC5282693; doi:10.1186/s12955-017-0604-1)
Supplement: Additional file 1: — Patient Centered Outcome Questionnaire. (DOCX 11 kb) [file 12955_2017_604_MOESM1_ESM.docx]

Additional file 1‐Patient Centered Outcome Questionnaire

MANY PEOPLE EXPERIENCE PAIN, FATIGUE (I.E., FEELING TIRED), EMOTIONAL DISTRESS

(E.G., WORRIES, FEELING SAD), AND INTERFERENCE WITH DAILY ACTIVITIES

(E.G., NOT BEING ABLE TO WORK OR DO HOUSEHOLD CHORES) AS A RESULT OF

THEIR MEDICAL CONDITION. WE WOULD LIKE TO UNDERSTAND HOW YOU HAVE

BEEN IMPACTED IN EACH OF THESE AREAS. WE WOULD ALSO LIKE TO LEARN MORE

ABOUT WHAT YOU WANT YOUR TREATMENT TO DO FOR YOU. _______________________________________________________________________________________

FIRST, WE WOULD LIKE TO KNOW YOUR USUAL LEVELS OF PAIN, FATIGUE, EMOTIONAL

DISTRESS, AND INTERFERENCE.

On a scale of 0 (none) to 100 (worst imaginable), please indicate your usual level (during the past

week) of . . .

• pain ______

• fatigue (or tiredness) ______

• emotional distress ______

• interference with daily activities ______ _______________________________________________________________________________________

NOW, WE WOULD LIKE TO LEARN ABOUT YOUR DESIRED LEVELS OF PAIN, FATIGUE,

Preliminary Results of Patient Defined Success Criteria for Individuals with Musculoskeletal Pain in

Outpatient Physical Therapy Settings

EMOTIONAL DISTRESS, AND INTERFERENCE. IN OTHER WORDS, WE WOULD LIKE

TO UNDERSTAND WHAT YOUR IDEAL TREATMENT OUTCOME WOULD BE.

On a scale of 0 (none) to 100 (worst imaginable), please indicate your desired level of . . .

• pain ______

• fatigue (or tiredness) ______

• emotional distress ______

• interference with daily activities ______ _______________________________________________________________________________________

PATIENTS UNDERSTANDABLY WANT THEIR TREATMENT TO RESULT IN DESIRED OR

IDEAL OUTCOMES LIKE YOU INDICATED ABOVE. UNFORTUNATELY, AVAILABLE

TREATMENTS DO NOT ALWAYS PRODUCE DESIRED OUTCOMES. THEREFORE, IT IS

IMPORTANT FOR US TO UNDERSTAND WHAT TREATMENT OUTCOMES YOU

WOULD CONSIDER SUCCESSFUL.

On a scale of 0 (none) to 100 (worst imaginable), please indicate the level each of these areas

would have to be at for you to consider treatment successful.

• pain ______

• fatigue (or tiredness) ______

• emotional distress ______

• interference with daily activities ______ _______________________________________________________________________________________

NOW, WE WOULD LIKE TO KNOW WHAT YOU EXPECT YOUR TREATMENT TO DO

FOR YOU.

Preliminary Results of Patient Defined Success Criteria for Individuals with Musculoskeletal Pain in

Outpatient Physical Therapy Settings

On a scale of 0 (none) to 100 (worst imaginable), please indicate the levels you expect following

treatment.

• pain ______

• fatigue (or tiredness) ______

• emotional distress ______

• interference with daily activities ______ _______________________________________________________________________________________

FINALLY, WE WOULD LIKE TO UNDERSTAND HOW IMPORTANT IT IS FOR YOU TO

SEE IMPROVEMENT IN YOUR PAIN, FATIGUE, EMOTIONAL DISTRESS, 1 AND INTERFERENCE FOLLOWING TREATMENT.

On a scale of 0 (not at all important) to 100 (most important), please indicate how important it is for you to see improvement in your . . .

• pain ______

• fatigue (or tiredness) ______

• emotional distress ______

• interference with daily activities ______
