# Supplementary material for: Patient-defined desired outcome, success criteria, and expectation in outpatient physical therapy: a longitudinal assessment
Source: Health Qual Life Outcomes. 2017 Jan 31;15:29. doi: 10.1186/s12955-017-0604-1 (PMC5282693; doi:10.1186/s12955-017-0604-1)
Supplement: Additional file 2: — Follow-up Patient Centered Outcome Questionnaire. (DOCX 14 kb) [file 12955_2017_604_MOESM2_ESM.docx]

Additional file 2- Follow-up Patient Centered Outcome Questionnaire

________________________________________________________________________

FIRST, WE WOULD LIKE TO KNOW YOUR **PRE-TREATMENT** LEVELS OF PAIN, FATIGUE, EMOTIONAL DISTRESS, AND INTERFERENCE.

On a scale of ***0 (none)* to *100 (worst imaginable)***, please estimate your pre-treatment level of …

• _pain _____

• _fatigue (or tiredness) _____

• _emotional distress _____

• _interference with daily activities _____

________________________________________________________________________

BASED ON YOUR PRE-TREATMENT LEVELS OF PAIN, FATIGUE, EMOTIONAL DISTRESS, AND INTERFERENCE, PLEASE INDICATE WHAT TREATMENT OUTCOMES YOU WOULD CONSIDER **SUCCESSFUL**.

On a scale of ***0 (none)* to *100 (worst imaginable)***, please indicate the level each of these areas would have to be at for you to consider treatment successful.

• _pain _____

• _fatigue (or tiredness) _____

• _emotional distress _____

• _interference with daily activities _____

________________________________________________________________________ NEXT, WE WOULD LIKE TO UNDERSTAND HOW **IMPORTANT** IT IS FOR YOU TO SEE IMPROVEMENT IN YOUR PAIN, FATIGUE, EMOTIONAL DISTRESS, AND INTERFERENCE.

On a scale of ***0 (not at all important)* to *100 (most important)***, please indicate how important it is for you to see improvement in your…

• _pain _____

• _fatigue (or tiredness) _____

• _emotional distress _____

• _interference with daily activities _____

**________________________________________________________________________**

FINALLY, WE WOULD LIKE TO KNOW YOUR **USUAL** LEVELS OF PAIN, FATIGUE, EMOTIONAL DISTRESS, AND INTERFERENCE **FOLLOWING TREATMENT**. IN OTHER WORDS, WHAT ARE YOUR CURRENT LEVELS OF PAIN, FATIGUE, EMOTIONAL DISTRESS, AND INTERFERENCE?

On a scale of ***0 (none)* to *100 (worst imaginable)***, please indicate your usual level (during the past week) of …

• _pain _____

• _fatigue (or tiredness) _____

• _emotional distress _____

• _interference with daily activities _____

ACCORDING TO OUR RECORDS, YOUR **PRE-TREATMENT** LEVELS OF PAIN, FATIGUE, EMOTIONAL DISTRESS, AND INTERFERENCE [ON A SCALE FROM 0 (NONE) TO 100 (WORST IMAGINABLE)] WERE AS FOLLOWS:

• _pain _____

• _fatigue (or tiredness) _____

• _emotional distress _____

• _interference with daily activities _____

________________________________________________________________________

NOW WE WOULD LIKE TO KNOW IF YOU HAVE **IMPROVED, STAYED THE SAME, OR WORSENED** SINCE THE START OF TREATMENT.

1. Has your pain improved, stayed the same, or worsened?

**Please choose one:**

_____ Improved (Please complete Part A below.)

_____ Stayed the same (Please move on to #2.)

_____ Worsened (Please complete Part B below.)

**Part A:** On a scale of ***0 (no improvement)* to *100 (complete improvement)***, please indicate how much your pain has improved _____

**Part B:** On a scale of ***0 (no worsening)* to *100 (complete worsening)***, please indicate how much your pain has worsened _____ (Please move on to #2.)

2. Has your fatigue (or tiredness) improved, stayed the same, or worsened?

**Please choose one:**

_____ Improved (Please complete Part A below.)

_____ Stayed the same (Please move on to #3.)

_____ Worsened (Please complete Part B below.)

**Part A:** On a scale of ***0 (no improvement)* to *100 (complete improvement)***, please indicate how much your fatigue has improved _____

**Part B:** On a scale of ***0 (no worsening)* to *100 (complete worsening)***, please indicate how much your fatigue has worsened _____

3. Has your emotional distress improved, stayed the same, or worsened?

**Please choose one:**

_____ Improved (Please complete Part A below.)

_____ Stayed the same (Please move on to #4.)

_____ Worsened (Please complete Part B below.)

**Part A:** On a scale of *0 (no improvement)* to *100 (complete improvement)*, please indicate how much your emotional distress has improved _____

**Part B**: On a scale of *0 (no worsening)* to *100 (complete worsening)*, please indicate how much your emotional distress has worsened _____

4. Has your interference improved, stayed the same, or worsened?

**Please choose one:**

_____ Improved (Please complete Part A below.)

_____ Stayed the same (Please move on to #5.)

_____ Worsened (Please complete Part B below.)

**Part A:** On a scale of ***0 (no improvement)* to *100 (complete improvement)***, please indicate how much your interference has improved _____

**Part B:** On a scale of ***0 (no worsening)* to *100 (complete worsening)***, please indicate how much your interference has worsened _____

5. Overall, have you improved, stayed the same, or worsened?

**Please choose one:**

_____ Improved (Please complete Part A below.)

_____ Stayed the same (Please move on to the next page.)

_____ Worsened (Please complete Part B below.)

**Part A:** On a scale of ***0 (no improvement)* to *100 (complete improvement)***, please indicate how much you have improved _____

**Part B:** On a scale of ***0 (no worsening)* to *100 (complete worsening)***, please indicate how much you have worsened _____ ____________________________________________________________________

FINALLY, WE WOULD LIKE TO LEARN WHETHER YOU WOULD CONSIDER YOUR TREATMENT **SUCCESSFUL**.

• _Was treatment of your pain successful?

**Please choose one:**

_____ Yes

_____ No

• _Was treatment of your fatigue (or tiredness) successful?

**Please choose one:**

_____ Yes

_____ No

• _Was treatment of your emotional distress successful?

**Please choose one:**

_____ Yes

_____ No

• _Was treatment of your interference with daily activities successful?

**Please choose one:**

_____ Yes

_____ No

• _Overall, was your treatment successful?

**Please choose one:**

_____ Yes

_____ No
